# Type of cancer treatment and cognitive symptoms in working cancer survivors: an 18-month follow-up study

**DOI:** 10.1007/s11764-019-00839-w

**Published:** 2020-01-15

**Authors:** Johanna K. Ehrenstein, Sander K.R. van Zon, Saskia F.A. Duijts, Boukje A.C. van Dijk, Heleen F. Dorland, Sanne B. Schagen, Ute Bültmann

**Affiliations:** 1 Department of Health Sciences, Community and Occupational Medicine, University of Groningen, University Medical Center Groningen, Hanzeplein 1, PO Box 30.001, 9700 RB Groningen, The Netherlands; 2 Department of Public and Occupational Health, Vrije Universiteit Amsterdam, Amsterdam UMC, Amsterdam Public Health Research Institute, Van der Boechorststraat 7, 1081 BT Amsterdam, The Netherlands; 3 Department of Research and Development, Comprehensive Cancer Center The Netherlands (IKNL), Godebaldkwartier, 3511 DT Utrecht, The Netherlands; 4 Department of Epidemiology, University of Groningen, University Medical Center Groningen, Hanzeplein 1, 9713 GZ Groningen, The Netherlands; 5 Division of Psychosocial Research and Epidemiology, The Netherlands Cancer Institute, Plesmanlaan 121, 1066 CX Amsterdam, The Netherlands; 6 Department of Psychology, University of Amsterdam, Nieuwe Achtergracht 129-B, 1018 WT Amsterdam, The Netherlands

**Keywords:** Cancer-related cognitive impairment, Type of cancer treatment, Employment, Psycho-oncology

## Abstract

**Purpose:**

Cognitive symptoms are reported to affect cancer survivors’ functioning at work. However, little is known about the type of cancer treatment and cognitive symptoms in working cancer survivors. We examined the longitudinal association between type of cancer treatment and cognitive symptoms in cancer survivors post return to work, and whether the course of cognitive symptoms over 18 months differed per type of cancer treatment.

**Methods:**

Data from the Dutch longitudinal “Work-Life after Cancer” study were used. The study population consisted of 330 working cancer survivors who completed questionnaires at baseline, and 6, 12, and 18 months follow-up. Cognitive symptoms were assessed with the cognitive symptom checklist-work and linked with cancer treatment data from the Netherlands Cancer Registry. Data were analyzed using generalized estimating equations.

**Results:**

Cancer survivors who received chemotherapy reported comparable memory symptom levels (*b*: − 2.3; 95% *CI* = − 7.1, 2.5) to those receiving locoregional treatment. Executive function symptom levels (*b*: − 4.1; 95% *CI* = − 7.8, − 0.4) were significantly lower for cancer survivors who received chemotherapy, compared with those receiving locoregional treatment. In cancer survivors who received other systemic therapy, memory (*b*: 0.4; 95% *CI* = 0.1, 0.7) and executive function symptom levels (*b*: 0.4; 95% *CI* = 0.0, 0.7) increased over time. In cancer survivors who received chemotherapy and locoregional treatment, memory and executive function symptom scores were persistent during the first 18 months after return to work.

**Conclusions:**

The contradictory finding that cancer patients receiving chemotherapy report fewer cognitive symptoms warrants further research.

**Implications for Cancer Survivors:**

Working cancer survivors may have cognitive symptom management needs irrespective of the type of cancer treatment they received.

**Electronic supplementary material:**

The online version of this article (10.1007/s11764-019-00839-w) contains supplementary material, which is available to authorized users.

## Introduction

Worldwide, 40–50% of all newly diagnosed cancer survivors are of working age and therefore, potentially part of the labor force [1, 2]. Overall, the percentage of cancer survivors able to return to work is 63.5% (range 24–94%) [3]. Despite advances in early detection and cancer treatment for cancer survivors of working age, cancer and its treatment can still result in a wide range of long-term physical and psychological problems, including fatigue, depression, and cognitive symptoms [4, 5]. Cognitive symptoms are frequently reported to affect cancer survivors’ functioning at work [6], and adverse consequences of even subtle cognitive symptoms may be profound. The findings of an integrative review suggest that cognitive symptoms are an essential factor for work ability, return to work, and work performance [6]. A recent cross-sectional study demonstrated that cognitive symptoms at work were associated with lower levels of quality, quantity, and timeliness of completed work among breast cancer survivors [7]. Patients who are cognitively affected may experience challenges on the job, especially in jobs that require peak performance in assimilating, processing, retaining, and utilizing information [8, 9]. The cognitive domains most likely to be negatively impacted are working memory and executive function [10]. Memory problems may influence a workers’ capacity to acquire the knowledge and skills necessary to carry out work-related functions [11] and executive function problems may diminish the planning and implementing of strategies. Optimal cognitive functioning is required in many non-manual (e.g., office work), and also in manual (e.g., construction work) occupations [12].

Cancer-related cognitive impairment (CRCI) is an important and prevalent problem for survivors, which can result from chemotherapy [13], but has also been associated with radiotherapy and surgery [10, 14]. Although several studies have assessed CRCI in a range of cancer populations, via neuropsychological testing and self-report assessments, there is a lack of longitudinal research examining occupationally active cancer survivors, and the cognitive symptoms they are dealing with while at work. In a previous study, Dorland et al. (2017) showed that working cancer survivors experienced more memory symptoms compared with executive function symptoms [15]. These symptoms persisted during 18 months follow-up. Assessing the survivor’s personal judgment of her/his ability to complete tasks in daily life is an important aspect of CRCI. The assessment provides insight into environmental and contextual factors that may facilitate or hinder performance.

Cognitive symptoms in the general population of cancer survivors (excluding central nervous system cancer) have mostly been attributed to the neurotoxic effects of chemotherapy [16, 17]. For example, Janelsins et al. (2018) showed that breast cancer patients had significantly worse cognitive symptoms from prechemotherapy to postchemotherapy and from prechemotherapy to 6-month follow-up and compared with healthy controls [18]. Particularly, cancer survivors treated with chemotherapy often report memory loss and executive dysfunction [13]. Although chemotherapy is a risk factor for developing CRCI, it is clearly not the only factor associated with this kind of impairment. Surgery [19, 20], other adjuvant therapies [21–23], and the cancer itself [3] may also lead to impairments due to an inflammatory response triggering neurotoxic cytokines. Further, CRCI has also been associated with psychological consequences of cancer and its treatment, including depression and fatigue [10, 14].

To date, no studies have examined the association between type of cancer treatment and self-reported cognitive symptoms in working cancer survivors, using a longitudinal study design. Understanding the association will be a valuable resource for occupationally active survivors’ health care providers in terms of treatment plans and guidance. Therefore, in the current study, we aimed to assess: [1] the longitudinal associations between type of cancer treatment and self-reported cognitive symptoms (memory and executive function) in working cancer survivors, and [2] to assess whether the course of cognitive symptoms over 18 months post return to work differed per treatment group. In previous studies, cognitive impairment in cancer patients has mostly been attributed to the neurotoxic effects of chemotherapy. Given this biomechanical explanation [8, 24], it is hypothesized that cognitive symptoms are more frequently present in cancer survivors who received chemotherapy than in cancer survivors who only received locoregional treatment (surgery and/or radiotherapy).

## Method

### Study design and population

The current study was conducted within the sampling frame of the Work-Life after Cancer (WOLICA) study, a longitudinal cohort study on health-related work functioning [25]. The total WOLICA cohort consisted of 384 cancer survivors who returned to work after being diagnosed and treated for cancer. Participants completed questionnaires at baseline (within 3 months after returning to work), and after 6, 12, and 18 months follow-up. The study population included consecutively recruited cancer survivors, aged 18–65 years. Eligible for participation were cancer survivors who [1] were treated with curative intent, and [2] returned to paid work for at least 12 h a week, according to the definition of the working population of Statistics Netherlands at the time that the data collection of the WOLICA study was initiated [26]. Participants were required to have a good command of written and spoken Dutch language. The recruiting occupational physicians informally appraised the Dutch language skills of potential participants. Cancer survivors who meet the following criteria were excluded: [1] having recurrent cancer because they represent a group with other/earlier experiences of cancer treatment, return to work, and prognosis, [2] having been treated with palliative intent/hospice care, and [3] having no paid employment for at least 1 year prior to the cancer diagnosis. Patients who started working after a period of being unemployed were not included to increase the homogeneity of the sample. Patients were included within 3 months after their return to work to directly assess the patients functioning after being sick listed and to increase the homogeneity of the sample*.*

Potentially eligible participants were identified and informed about the study by their occupational physician (OP) during a regular visit in the return to work process. The OPs worked at three national Occupational Health Services (OHS) in the Netherlands, covering about 33% of all workers in the Netherlands. Between March 2013 and July 2015, 516 cancer survivors were contacted for participation in the WOLICA study. Of those contacted, 53 were excluded for different reasons (i.e., not eligible, could not be reached, died), resulting in 463 eligible participants. The response rate for the baseline questionnaire was 84% (387 out of 463). Further, three participants were excluded after completing the baseline questionnaire because their return to work was longer than 3 months ago, resulting in the 384 participants of the WOLICA study. Participants completed the questionnaires at home. Questionnaires were returned by mail or completed online. More detailed information about the recruitment procedure can be found in the study of Dorland et al. (2017) [25].

Questionnaire data of 371 cancer survivors were linked to the Netherlands Cancer Registry (NCR), a large prospective registry of all incident cases of cancer in the Netherlands, with nationwide coverage since 1989. Thirteen cases from the WOLICA study could not be linked to the NCR: three persons were diagnosed and treated outside the Netherlands and were therefore not included in the NCR; for the other 10 cases, the reason is unknown. Those cancer survivors were not included in the study. The record linkage with the NCR also showed that 41 cancer survivors had a prior cancer diagnosis, and were therefore excluded from the analysis, resulting in a final number of 330 survivors in the current study.

Ethical approval for the WOLICA study was granted by The Medical Ethical Committee of the University Medical Center Groningen (UMCG) (METC number: M12.125242). Informed consent to participate was obtained from all participants prior to the study.

## Measurements

### Work-related cognitive symptoms

Work-related cognitive symptoms were assessed at baseline and at 6, 12, and 18 months follow-up using the cognitive symptom checklist-work (Dutch Version) (CSC-W) [10]. The CSC-W is a reliable (19 items, *α* = 0.95) and valid self-reported measure of work-related cognitive symptoms in occupational active cancer survivors [10]. The CSC-W comprises two subscales and measures self-reported *memory symptoms* (8 items *α* = 0.93) and *executive function symptoms* (11 items *α* = 0.94). The memory symptoms subscale measures the frequency of symptoms experienced by cancer survivors with remembering, e.g., “I have difficulty remembering the content of telephone conversations.” The executive function symptoms subscale measures the frequency of symptoms experienced by cancer survivors when using new information, e.g., “I have difficulty completing all steps of a task or activity” or “I have difficulty to understand that specific tasks belong to a larger whole.” All items were rated on a Likert-scale that ranges from 0 (never) to 4 (always). The total score and subscale scores were obtained by summing the scores on each item, divided by the number of items. The average score is multiplied by 25*.* Scores range from 0 to 100, with higher scores indicating more cognitive symptoms. When 20% or more of the items are missing*,* the scale score was set to missing [10].

### Type of cancer treatment

Based on data from the NCR, the following treatment categories were distinguished: [1] “locoregional” treatment (i.e., surgery and/or radiotherapy), [2] “chemotherapy” using chemotherapy, exclusively or in combination with another type of treatment, and [3] other “systemic” therapy using hormonal therapy or targeted therapy, exclusively or in combination with surgery and/or radiotherapy.

### Potential confounding factors

The selection of potential confounding factors was based on the conceptual model of cancer-related cognitive impairment (“Chemotherapy-Related Change in Cognitive Function”) [27] and included sociodemographic factors, clinical and treatment-related factors, and psychological symptoms.

*Sociodemographic factors* comprised gender (male; female), age (in years), and education at baseline. Education was classified as [1] low, i.e., primary, junior secondary vocational, and junior general secondary education; [2] medium, i.e., senior secondary vocational education and senior general secondary education, and [3] high, i.e., higher professional education, college, and university.

*Clinical and treatment-related factors* were obtained through record linkage with the NCR and included: still undergoing treatment at baseline (yes; no), time between cancer diagnosis and return to work for at least 12 h per week (in months) and extent of disease. The “extent of disease” cancer staging system based on the National Cancer Institute Surveillance, Epidemiology and End Results (SEER) [28] program was used. It permits staging of patients in a wide spectrum of cancer populations. The cancer was classified into four categories: [1] localized, i.e., the cancer is limited to the place where it started, with no sign that it has spread, [2] regional, i.e., the cancer has spread to nearby lymph nodes, tissues, or organs, [3] distant, i.e., the cancer has spread to distant parts of the body, and [4] unknown, i.e., there is not enough information to determine the stage.

*Psychological symptoms* included depressive symptoms and fatigue as time-varying covariates. Depressive symptoms were assessed using the patient health questionnaire-9 (PHQ-9) [29, 30], a 9-item multiple-choice self-report inventory screening the presence and severity of depression. Response options range from 0 (not at all) to 3 (nearly every day). Total scores range from 0 to 27, with higher scores indicating more depressive symptoms. Fatigue was assessed using the “fatigue severity” subscale of the checklist individual strength (CIS-8) [31] an 8-item multiple-choice questionnaire. Response options range from 1 (yes, that is true) to 7 (no, that is not true). Total scores were obtained by summing the scores on each item*.* Total scores range from 8 to 56, with higher scores indicating a higher degree of fatigue.

### Statistical analysis

Descriptive data analyses were performed for the total study sample and stratified by type of cancer treatment. Linear generalized estimating equations (GEE) with exchangeable working correlation structure were used to examine the longitudinal association between type of cancer treatment and self-reported cognitive symptoms in working cancer survivors. The GEE model allows analyzing the variables of the model at different time points simultaneously. The analysis takes into account the intra-individual correlations between the observations [15]. A stepwise approach to examine the longitudinal association between type of cancer treatment and memory and executive function symptoms was used. Five models were fitted for memory and executive function: Model 1 was an unconditional growth model and included time as a categorical variable. Model 2 additionally included type of cancer treatment. Model 3 additionally included age, gender, and education. Model 4 additionally included treatment completion, time between diagnosis and return to work in months, and extent of disease. Finally, model 5 additionally included depression and fatigue.

Linear GEE were conducted to assess whether the course of cognitive symptoms over 18 months after return to work differed per treatment type. The same models as in the main analysis were fitted, except that time in months was included as a continuous variable, and that an interaction term between type of cancer treatment and time (in months) was included. Regression coefficients with corresponding 95% confidence intervals (CIs) were reported. Available data and the percentage of person-measurement observations for each model were reported for memory symptoms and executive function symptoms. Statistical analyses were conducted with the statistical package for social sciences (SPSS) version 24.

## Results

### Baseline characteristics

Of the 330 cancer survivors, 62.7% were women (Table 1). The mean age at baseline was 50.6 years (*SD* = 8.7). Seventy-nine percent of the patients were living with a partner, and 38.9% had a high educational level. Fourteen different types of cancer were identified in the participating survivors (Table 2). Nearly half of the participants (47.3%) had breast cancer, followed by male reproductive cancers (10.3%) and hematological cancer (9.1%). More than half of the patients (59.9%) received chemotherapy. Thirty-one percent of the patients received locoregional treatment. Two-third (63.2%) of the cancer survivors had completed their treatment at baseline. Tables 1 and 2 show baseline characteristics, including sociodemographic factors and clinical factors, stratified by type of cancer treatment. The retention rates were high with 302 (91% of 330) participants completing the questionnaire after 6 months, 280 (85% of 330) participants completing the questionnaire after 12 months, and 267 (81% of 330) participants completing the questionnaire after 18 months.

**Table 1:** Participant characteristics at baseline (*n* = 330) stratified by treatment type

	Total (*n* = 330)	Locoregional treatment (*n* = 100)	Chemotherapy (*n* = 194)	Other systemic therapy (*n* = 30)
Age in years, *M* ± *SD*	50.6 ± 8.7	52.5 (8.1)	48.9 (8.9)	55.3 (5.6)
Gender, *n* (%)				
Male	123 (37.3)	53 (53.0)	54 (27.8)	12 (40.0)
Female	207 (62.7)	47 (47.0)	140 (72.2)	18 (60.0)
Marital status, *n* (%)				
Married/cohabitating	258 (78.4)	80 (80.0)	151 (78.2)	21 (70.0)
Single/divorced/separated	71 (21.6)	20 (20.0)	42 (21.8)	9 (30.0)
Education, *n* (%)				
Low	88 (26.7)	30 (30.0)	45 (23.3)	13 (43.3)
Medium	113 (34.3)	32 (32.0)	71 (36.8)	6 (20.0)
High	128 (38.9)	38 (38.0)	77 (39.9)	11 (36.7)
Type of job, *n* (%)				
Manual	40 (12.2)	10 (10.0)	26 (13.5)	4 (13.3)
Non-manual	189 (56.8)	56 (56.0)	115 (59.6)	13 (43.3)
Both manual and non-manual	102 (31.0)	34 (34.0)	52 (26.9)	13 (43.3)

Note: *M*, mean; *SD*, standard deviation

**Table 2:** Diagnosis and treatment characteristics at baseline (*n* = 330) stratified by treatment type

	Total (*n* = 330)	Locoregional treatment (*n* = 100)	Chemotherapy (*n* = 194)	Other systemic therapy (*n* = 30)
Tumor diagnosis, *n* (%)				
Breast cancer	156 (47.3)	23 (23.0)	115 (59.3)	17 (56.7)
Male reproductive cancers	34 (10.3)	18 (18.0)	6 (3.1)	9 (30.0)
Hematologic cancer	30 (9.1)	1 (1.0)	25 (12.9)	1 (3.3)
Gastrointestinal cancer	26 (7.9)	8 (8.0)	16 (8.2)	1 (3.3)
Colon cancer	23 (7.0)	9 (9.0)	14 (7.2)	
Skin cancer	13 (3.9)	13 (13.0)		
Gynecological cancer	11 (3.3)	4 (4.0)	7 (3.6)	
Head and neck cancer	10 (3.0)	8 (8.0)	1 (0.5)	1 (3.3)
Lung cancer	10 (3.0)	1 (1.0)	8 (4.1)	1 (3.3)
Urological cancer	10 (3.0)	10 (10.0)		
Endocrine cancer	3 (0.9)	3 (3.0)		
Bone cartilage and soft tissue cancer	2 (0.6)	1 (1.0)	1 (0.5)	
Central nervous system cancer	1 (0.3)		1 (0.5)	
Eye cancer	1 (0.3)	1 (1.0)		
Extent of disease, *n* (%)				
Local	109 (33.0)	48 (48.0)	49 (25.3)	12 (40.0)
Reginal	93 (28.2)	14 (14.0)	75 (38.7)	4 (13.3)
Distant	10 (3.0)		7 (3.6)	3 (10.0)
Unknown	118 (35.8)	38 (38.0)	63 (32.5)	11 (36.7)
Treatment completed, *n* (%)				
Yes	208 (63.2)	94 (94.9)	104 (53.6)	7 (23.3)
No	121 (36.8)	5 (5.1)	90 (46.4)	23 (76.7)
Time diagnosis to RTW, ^a^ *M* ± *SD*	7.4 ± 6.4	5.6 ± 7.4	8.6 ± 5.5	4.5 ± 5.4
Memory symptoms,^1^ *M* ± *SD*	32.1 ± 19.6	32.6 ± 18.9	31.7 ± 20.1	35.9 ± 18.7
Executive function symptoms,^2^ *M* ± *SD*	19.3 ± 15.9	20.4 ± 14.9	18.0 ± 16.0	24.8 ± 17.5
Depressive symptoms, *M* ± *SD*	4.6 ± 3.6	5.0 ± 3.9	4.3 ± 3.3	5.3 ± 4.1
Fatigue, M ± SD	30.1 ± 11.4	30.6 ± 11.7	29.3 ± 11.2	33.6 ± 10.7

Note: *M*, mean; *SD*, standard deviation;^. 1^For the comparison between treatment types *p* = 0.564; ^2^ For the comparison between treatment types *p* = 0.083

## Association between type of cancer treatment and work-related cognitive symptoms

### Memory

Memory symptoms remained stable between baseline and 18 months (Table 3, model 1, *n* = 1126 person measurement observations, 85.3%). Adding type of cancer treatment in model 2 showed that there were no differences over time between cancer survivors who received chemotherapy compared with those who received locoregional treatment. Cancer survivors who received other systemic therapy, reported higher symptoms in working memory over time compared with those treated with locoregional treatment. The associations remained unchanged after additional adjustments for sociodemographic factors (model 3), clinical and treatment-related factors (model 4), and psychological symptoms (model 5). In the final model, depression and fatigue were positively associated with symptoms in memory over 18 months (model 5).

**Table 3:** Longitudinal associations between treatment-related factors and memory symptoms in 330 cancer survivors with different cancer diagnoses

	Model 1	Model 2	Model 3	Model 4	Model 5
Intercept	32.10 (29.97, 34.22)***	31.38 (27.85, 34.90)***	30.33 (17.39, 43.27)***	31.98 (17.71, 46.24)***	24.99 (10.80, 39.17)***
Time					
Baseline	Ref	Ref	Ref	Ref	Ref
6 months	− 1.00 (− 2.54, 0.55)	− 1.08 (− 2.65, 0.49)	− 1.08 (− 2.66, 0.50)	− 1.00 (− 2.61, 0.60)	− 0.19 (− 1.81, 1.44)
12 months	− 0.75 (− 2.27, 0.77)	− 0.68 (− 2.22, 0.85)	− 0.69 (− 2.23, 0.86)	−0. 56 (− 2.14, 1.02)	0.07 (− 1.52, 1.66)
18 months	− 1.60 (− 3.29, 0.09)	− 1.49 (− 3.25, 0.27)	− 1.51 (− 3.27, 0.26)	− 1.55 (− 3.33, 0.24)	− 1.08 (− 2.85, 0.68)
Type of cancer treatment					
Locoregional treatment		Ref	Ref	Ref	Ref
Chemotherapy		0.09 (− 4.23, 4.40)	− 1.77 (− 6.19, 2.65)	− 2.81 (− 7.80, 2.19)	− 2.30 (− 7.11, 2.50)
Other systemic therapy^1^		8.37 (0.94, 15.79)*	8.30 (1.21, 15.40)*	11.55 (3.32, 19.78)**	11.14 (3.06, 19.21)**
Gender					
Male			Ref	Ref	Ref
Female			5.27 (1.10. 9.45)*	4.53 (− 0.35, 9.42)	4.69 (0.00, 9.38)
Age			− 0.05 (− 0.28, 0.19)	− 0.08 (− 0.31, 0.16)	− 0.04 (− 0.27. 0.19)
Education					
High			Ref	Ref	Ref
Medium			4.82 (0.23, 9.41)*	4.62 (− 0.03, 9.28)	4.47 (− 0.03, 8.97)
Low			− 1.52 (− 6.36, 3.32)	− 1.51 (− 6.39, 3.37)	− 1.43 (− 6.15, 3.30)
Treatment completed					
Yes				Ref	Ref
No				− 1.27 (− 6.13, 3.59)	− 1.39(− 6.11, 3.33)
Time diagnosis to RTW^a^				− 0.27 (− 0.58, 0.05)	− 0.23 (− 0.53, 0.08)
Extent of disease					
Local				Ref	Ref
Regional				− 0.72 (− 5.87, 4.43)	− 0.70 (− 5.66, 4.26)
Distant				− 5.10 (− 16.25, 6.04)	− 5.46 (− 16.02, 5.11)
Unknown				− 1.77 (− 7.14, 3.60)	− 2.01 (− 7.19, 3.17)
Depressive symptoms^b^					0.11 (0.03, 0.20)**
Fatigue^c^					0.16 (0.06, 0.26)**

Note: *p value < 0.05; ** *p* value < 0.01; *** *p* value < 0.001. Intercept, slopes, and 95% confidence intervals were presented. ^1^Other “systemic” therapy using hormonal therapy or targeted therapy, exclusively or in combination with surgery and/or radiotherapy. *RTW*, return to work; ^a^ in months; ^b^ range 0–28 ; ^c^ range 8–56. Model 1 included *n* = 1126 (85.3%); Model 2 *n* = 1109 (84.0%); Model 3 *n* = 1102 (83.5%); Model 4 *n* = 1063 (80.5%); Model 5 *n* = 1063 (80.5%) of the 1320 possible person-measurement observations

### Executive function

Executive function symptoms remained stable between baseline and 18 months (Table 4, model 1, 1084 person measurement observations, 82.1%). Adding type of cancer treatment in model 2 showed that differences between cancer survivors who received chemotherapy compared with those who received locoregional treatment were not significant over time. These associations remained unchanged after adjustment for sociodemographic factors (model 3). After additional adjustments for clinical and treatment-related factors and psychological symptoms (models 4 and 5), executive function symptom scores were significantly lower for cancer survivors who received chemotherapy compared with those who received locoregional treatment. Cancer survivors who received other systemic therapy reported more symptoms in executive function compared with those who only received locoregional treatment. These associations remained statistically significant after adjustment for sociodemographic factors (model 3) and after adjustments for clinical and treatment-related and psychological factors (models 4 and 5). In the final model, time between diagnosis and return to work in months was negatively associated with symptom scores in executive function. In addition, fatigue was positively associated with symptoms scores in executive function over 18 months (model 5).

**Table 4:** Longitudinal associations between treatment-related factors and executive function symptoms in 330 cancer survivors with different cancer diagnoses

	Model 1	Model 2	Model 3	Model 4	Model 5
Intercept	19.14 (17.37, 20.90)***	19.60 (16.79, 22.40)***	12.71 (3.30, 22.11)**	16.04 (5.16, 26.93)**	10.13 (− 0.80, 21.05)
Time					
Baseline	Ref	Ref	Ref	Ref	Ref
6 months	− 0.21 (− 1.79, 1.38)	− 0.15 (− 1.72, 1.41)	− 0.19 (− 1.77, 1.39)	− 0.40 (− 1.95, 1.16)	0.25 (− 1.30, 1.79)
12 months	− 0.04 (− 1.58, 1.50)	0.03 (− 1.54, 1.60)	0.11 (− 1.47, 1.68)	0.07 (− 1.54, 1.67)	0.54 (− 1.06, 2.14)
18 months	0.11 (− 1.44, 1.66)	0.20 (− 1.37, 1.78)	0.22 (− 1.36, 1.80)	0.18 (− 1.42, 1.78)	0.55 (− 1.04, 2.15)
Type of cancer treatment					
Locoregional treatment		Ref	Ref	Ref	Ref
Chemotherapy		− 1.94 (− 5.33, 1.46)	− 2.32 (− 5.82, 1.19)	− 4.52 (− 8.34, − 0.70)*	− 4.05 (− 7.75, − 0.35)*
Other systemic therapy^1^		7.83 (1.48, 14.17)*	7.78 (1.49, 14.06)*	8.35 (1.30, 15.40)*	7.97 (0.95, 14.99)*
Gender					
Male			Ref	Ref	Ref
Female			2.34 (− 0.88, 5.56)	0.41 (− 3.39, 4.20)	0.50 (− 3.17, 4.17)
Age			0.08 (− 0.10, 0.25)	0.02 (− 0.16, 0.20)	0.05 (− 0.12, 0.23)
Education					
High			Ref	Ref	Ref
Medium			4.66 (1.07, 8.25)*	4.27 (0.61, 7.94)*	4.12 (0.57,7.67)*
Low			0.84 (− 3.19, 4.86)	0.85 (− 3.20, 4.90)	1.02 (− 2.95, 4.99)
Treatment completed					
Yes				Ref	Ref
No				1.15 (− 2.80, 5.11)	1.05 (− 2.84, 4.95)
Time diagnosis to RTW ^a^				− 0.29 (− 0.53, − 0.06)*	− 0.26 (− 0.49, − 0.03)*
Extent of disease					
Local				Ref	Ref
Regional				1.35 (− 2.86, 5.57)	1.35 (− 2.76, 5.46)
Distant				− 0.97 (− 11.43, 9.49)	− 1.27 (− 11.21, 8.66)
Unknown				− 2.53 (− 6.82, 1.77)	− 2.72 (− 6.88, 1.45)
Depressive symptoms ^b^					0.08 (− 0.03, 0.18)
Fatigue ^c^					0.13 (0.03, 0.24)*

Note: * *p* value < 0.05; ** *p* value < 0.01; *** *p* value < 0.001. Intercept, slopes, and 95% confidence intervals were presented. ^1^Other “systemic” therapy using hormonal therapy or targeted therapy, exclusively or in combination with surgery and/or radiotherapy. *RTW*, return to work; ^a^ in months; ^b^ range 0–28; ^c^ range 8–56. Model 1 included *n* = 1084 (82.1%); Model 2 *n* = 1067 (80.8%); Model 3 *n* = 1060 (80.3%); Model 4 *n* = 1024 (77.6%.); Model 5 *n* = 1023 (77.5%) of the 1320 possible person-measurement observations

### Course of cognitive symptoms over 18 months per treatment type

The interaction term between type of cancer treatment and time was statistically significant for memory symptoms. Memory symptom scores of cancer survivors who received other systemic therapy increased by 0.37 points per month (*CI* = 0.05, 0.70) compared with cancer survivors who received locoregional treatment, indicating more memory symptoms (Supplementary Table 1). Similarly, the interaction term between type of cancer treatment and time for executive function symptom scores was statistically significant. Executive function symptom scores of cancer survivors who received other systemic therapy increased by 0.35 points per month (*CI* = 0.01, 0.70) compared with cancer survivors who received locoregional treatment, indicating more executive function symptoms (Supplementary Table 1). These interaction terms remained statistically significant after adjustment for sociodemographic, clinical and treatment-related, and psychological factors. There were no significant interactions between locoregional treatment and time, and chemotherapy and time for memory symptoms and executive function symptoms.

## Discussion

This study is, to the best of our knowledge, the first longitudinal study to examine type of cancer treatment and cognitive symptoms in working cancer survivors. Cancer survivors who received chemotherapy and cancer survivors who received locoregional treatment had comparable levels of memory symptom scores. The level of symptoms regarding executive function was significantly lower for cancer survivors who received chemotherapy, compared with those receiving locoregional treatment. Cancer survivors who received other systemic therapy reported more symptoms in memory and executive function, compared with those receiving locoregional treatment. In cancer survivors who received other systemic therapy, memory and executive function symptom scores increased over time compared with cancer survivors who received locoregional treatment. In cancer survivors who received chemotherapy, and cancer survivors who received locoregional treatment, memory and executive function symptom scores remained stable, but persistent, during the first 18 months after return to work.

### Interpretation of the findings

In a previous review, including studies that followed cancer survivors up to 1–2 years post-treatment, it was shown that cognitive symptoms can arise during cancer treatment and can persist up to several years after completion of treatment [13]. In line with these findings, this study showed that memory and executive function symptoms in cancer survivors were continuously present, during the first 18 months after return to work.

The finding that cancer survivors who receive chemotherapy, had comparable levels of memory symptoms and lower levels of executive function symptoms than cancer survivors who received locoregional treatment is not in line with previous studies. Previous studies show that chemotherapy is the main, albeit not the only driver of CRCI [13]. It is important to consider that data derived from the self-reported CSC-W in WOLICA, might not be directly comparable to neuropsychological assessments. No longitudinal studies have used self-reported questionnaires to compare levels of cognitive symptoms in cancer survivors, treated with chemotherapy, to levels in those who received other treatments. Nevertheless, the finding that more intensively treated cancer survivors do not have more cognitive complaints, and have even lower symptoms, compared with those who received locoregional treatment is surprising. Moreover, Janelsins et al. (2017) showed that breast cancer patients had significantly higher self-reported cognitive symptoms from prechemotherapy to postchemotherapy as well as from prechemotherapy to 6-month follow-up [18]. The current findings might be explained by the fact that this sample consisted of occupationally active cancer survivors, and that chemotherapy is negatively associated with return to work [32, 33]. Notably, cancer survivors exposed to chemotherapy or a combination of therapies (e.g., surgery, radiotherapy, and chemotherapy) have a fourfold higher risk of not returning to work in the first (or even the three) year(s) following treatment, compared with cancer survivors who only had surgery or one type of treatment [32]. In line with this, it can be reasoned that the cancer survivors in this study, who are exposed to chemotherapy and are currently working, may represent a high functioning subset of cancer survivors treated with chemotherapy.

It was further found that the course of cognitive symptoms differed per type of cancer treatment. Memory and executive function symptom scores increased over time in cancer survivors who received other systemic therapy compared with cancer survivors who received locoregional treatment. An explanation may be that in those who received other systemic therapy, 76% were still on active treatment at baseline. This percentage is much higher than the percentage of survivors on active treatment in those receiving chemotherapy or locoregional treatment. In addition, the sample size of this group is comparably small.

### Strengths and limitations

A strength of this study is that a longitudinal design was used with repeated measurements of cognitive symptoms in working cancer survivors at baseline, 6, 12, and 18 months after return to work. Data of all four measurement points were available for the majority (81%) of participants. A validated measure of work-related cognitive symptoms was employed, i.e., the CSC-W [10], and linked with objective data on clinical factors from the NCR. Differences between chemotherapy-treated and non-chemotherapy-treated patients that might impact the reporting of cognitive symptoms are accounted for, including sociodemographic factors, clinical and treatment-related factors, and psychological symptoms. However, some unmeasured confounding might be present to at least some degree.

Also, some limitations have to be acknowledged. While the CSC-W takes into account the work environment, a combination of both self-reported and performance-based measures, i.e., neuropsychological tests, would be preferable. The combination of both self-reported and performance-based measures would enable the investigation of the independent and combined contributions of self-reported and performance-based measures to the risk of work-related cognitive symptoms. Also, for the CSC-W, there are no cut-offs available, making it difficult to determine whether an individual’s symptom level of cognitive functioning is a clinically relevant sign. Another limitation might concern selection bias. Potential participants were identified and informed about the current study by their OP during a regular visit in the return to work process. Possibly, the sample might be biased towards patients who resumed work after cancer diagnosis and treatment with better cognitive functioning, while patients with poorer outcomes might be underrepresented. At baseline, one-third of cancer patients were still undergoing treatment. Although treatment completion at baseline was controlled for, we did not adjust for possible treatment completion during follow-up, as this information was not available at the time of the analyses. Because cognitive effects of hormone therapy for breast cancer and prostate cancer may occur, given the critical role of hormones in the brain [13, 34], future studies may also control for when these individuals completed treatment.

### Implications for practice and research

The findings may have implications for the management of cognitive symptoms of cancer survivors at work. Awareness that cognitive symptoms may persist after return to work should be increased in cancer patients, employers, colleagues, (occupational) health care professionals, and the society as a whole. Assessment of cognitive symptoms in working cancer patients is important to provide accurate information on the occurrence of cognitive symptoms in working cancer survivors, as well as assistance with symptom management. The findings may help to inform policy and practice to act upon cognitive limitations in working cancer survivors. Occupational health care practitioners, employers, general practitioners, policymakers and other relevant stakeholders should lay out priorities and target efforts to aid working cancer survivors. The CSC-W has been included in the Guideline “Cancer and Work” of the Netherlands Society of Occupational Medicine [35] and this study may provide additional information for occupational physicians.

Because the reporting of cognitive symptoms may be rooted in part in psychological states such as depression and fatigue, future research should focus on the interrelation between treatment, cognitive functioning, and psychological symptoms. In addition, a combination of both self-reported and performance-based measures of cognitive functioning would be preferred, as studies based on neuropsychological assessment in working cancer survivors are lacking [8]. Also, it would be informative to compare cancer survivors who returned to work to those who did not, with respect to their cognitive functioning.

## Conclusion

Symptom scores in memory were comparable for cancer survivors who were treated with chemotherapy, and those who received locoregional treatment. Executive function symptom scores were significantly lower for cancer survivors treated with chemotherapy, compared with those who received locoregional treatment. Cancer survivors treated with other systemic therapy decreased more regarding memory and executive function once back at work compared with those treated with chemotherapy and locoregional treatment. Because employment is an important aspect of rehabilitation, working cancer survivors who experience cognitive symptoms should be identified, irrespective of their treatment type. The current study may help increase awareness and improve recognition and management of work-related cognitive symptoms by (occupational) health care professionals.

### Electronic supplementary material

ESM 1 (DOCX 37.2 kb)
