# Supplementary material for: Type of cancer treatment and cognitive symptoms in working cancer survivors: an 18-month follow-up study
Source: J Cancer Surviv. 2020 Jan 15;14(2):158–67. doi: 10.1007/s11764-019-00839-w (PMC7182617; doi:10.1007/s11764-019-00839-w)
Supplement: Supplementary file 1 — (DOCX 37.2 kb) [file 11764_2019_839_MOESM1_ESM.docx]

**Type of cancer treatment and cognitive symptoms in working cancer survivors:**

**an 18-month follow-up study**

**Journal of Cancer Survivorship**

Authors: Johanna K. Ehrenstein^1^, Sander K.R. van Zon^1^, Saskia F.A. Duijts^2,3^, Boukje A.C. van Dijk^3,4^, Heleen F. Dorland^1^, Sanne B. Schagen^5,6^, Ute Bültmann^1^.

**Name, City, and Affiliation of all authors:**

^1^ University of Groningen, University Medical Center Groningen, Department of Health Sciences, Community and Occupational Medicine, Hanzeplein 1, PO Box 30.001, 9700 RB, Groningen, The Netherlands

^2^ Vrije Universiteit Amsterdam, Amsterdam UMC, Amsterdam Public Health Research Institute, Department of Public and Occupational Health, Van der Boechorststraat 7, 1081 BT, Amsterdam, The Netherlands

^3^Comprehensive Cancer Center The Netherlands (IKNL), Department of Research and Development, Godebaldkwartier, 3511 DT, Utrecht, The Netherlands

^4^ University of Groningen, University Medical Center Groningen, Department of Epidemiology, Hanzeplein 1, 9713 GZ, Groningen, Netherlands

^5^ The Netherlands Cancer Institute, Division of Psychosocial Research and Epidemiology, Plesmanlaan 121, 1066 CX, Amsterdam, The Netherlands

^6^ University of Amsterdam, Department of Psychology, Nieuwe Achtergracht 129-B,

1018 WT, Amsterdam, The Netherlands

**Corresponding author:**

Johanna K. Ehrenstein, MSc

University of Groningen, University Medical Center Groningen

Department of Health Sciences, Community and Occupational Medicine

Hanzeplein 1, PO Box 30.001, 9700 RB

9713 AV Groningen

The Netherlands

Telephone: +31 645514967

E-mail: [j.k.ehrenstein@umcg.nl](mailto:j.k.ehrenstein@umcg.nl)

| **Supplementary Table 1** Longitudinal associations between treatment-related factors and working memory symptoms in 330 cancer patients with different cancer diagnoses | | | | | |
| --- | --- | --- | --- | --- | --- |
|  | **Model 1** | **Model 2** | **Model 3** | **Model 4** | **Model 5** |
| **Intercept** | 31.94 (29.86, 34.02)*** | 31.75 (28.14, 35.35)*** | 30.67 (17.71, 43.62)*** | 32.30 (18.02, 46.59)**** | 25.48 (11.24, 39.72)*** |
| **Time** | -0.08 (-0.17, 0.01) | -0.14 (-0.30, 0.02) | -0.14 (-0.30, 0.02) | -0.14 (-0.30, 0.02) | -0.10 (-0.26, 0.06) |
| **Type of cancer treatment** |  |  |  |  |  |
| Locoregional treatment |  | Ref | Ref | Ref | Ref |
| Chemotherapy |  | -0.41 (-4.96, 4.15) | -2.28 (-6.87, 2.32) | -3.24 (-8.39, 1.92) | -2.51 (-7.52, 2.50) |
| Other systemic therapy^1^ |  | 5.52 (-1.77, 12.82) | 5.45 (-1.40, 12.30) | 8.44 (0.53, 16.36)* | 8.21 (0.53, 15.89)* |
| **Type of cancer treatment * time** |  |  |  |  |  |
| Local treatment * time |  | Ref | Ref | Ref | Ref |
| Chemotherapy * time |  | 0.06 (-0.14, 0.27) | 0.07 (-0.14, 0.27) | 0.06 (-0.15, 0.26) | 0.03 (-0.17, 0.23) |
| Other systemic therapy^1^ * time |  | 0.36 (0.03, 0.69)* | 0.36 (0.03, 0.70)* | 0.40 (0.05, 0.75)* | 0.37 (0.05, 0.70)* |
| **Gender** |  |  |  |  |  |
| Male |  |  | Ref | Ref | Ref |
| Female |  |  | 5.26 (1.09, 9.43)* | 4.56 (-0.34 9.45) | 4.70 (0.00, 9.40) |
| **Age** |  |  | -0.04 (-0.28, 0.19) | -0.08 (-0.31, 0.16) | -0.04 (-0.27, 0.19) |
| **Education** |  |  |  |  |  |
| High |  |  | Ref | Ref | Ref |
| Medium |  |  | 4.88 (0.28, 9.47)* | 4.67 (0.01, 9.32)* | 4.50 (-0.01, 9.00) |
| Low |  |  | -1.52 (-6.36, 3.33) | -1.53 (6.41, 3.35) | -1.45 (-6.17, 3.28) |
| **Treatment completed** |  |  |  |  |  |
| Yes |  |  |  | Ref | Ref |
| No |  |  |  | -1.31 (-6.19, 3.57) | -1.42 (-6.16, 3.32) |
| **Time diagnosis to RTW ^a^** |  |  |  | -0.26 (-0.58, 0.05) | -0.23 (-0.53, 0.08) |
| **Extent of disease** |  |  |  |  |  |
| Local |  |  |  | Ref | Ref |
| Regional |  |  |  | -0.73 (-5.87, 4.42) | -0.72 (-5.68, 4.24) |
| Distant |  |  |  | -5.17 (-16.37, 6.02) | -5.60 (-16.25, 5.05) |
| Unknown |  |  |  | -1.72 (-7.09, 3.66) | -1.99(-7.17, 3.20) |
| **Depressive symptoms ^b^** |  |  |  |  | 0.11 (0.03, 0.19)** |
| **Fatigue ^c^** |  |  |  |  | 0.16 (0.05, 0.26)** |
| Note: * *p* value < 0.05; ** *p* value < 0.01; *** *p* value < 0.001. Intercept, slopes and 95% confidence intervals were presented. ^1^ Other “systemic” therapy using hormonal therapy or targeted therapy, exclusively or in combination with surgery and/or radiotherapy. *RTW*, return to work; ^a^ in months; ^b^ range 0–28 ^c^range 8–56. Model 1 included *n* = 1126 (85.3%); Model 2 *n* = 1109 (84.0%); Model 3 *n* = 1102 (83.5%); Model 4 *n* = 1063 (80.5%.); Model 5 *n* = 1062 (80.5%) of the 1320 possible person-measurement observations | | | | | |

| **Supplementary Table 2**  Longitudinal associations between treatment-related factors and executive function symptoms in 330 cancer patients with different cancer diagnoses | | | | | |
| --- | --- | --- | --- | --- | --- |
|  | **Model 1** | **Model 2** | **Model 3** | **Model 4** | **Model 5** |
| **Intercept** | 19.03 (17.41, 20.65)*** | 20.27 (17.50, 23.05)*** | 13.31 (3.88, 22.74)** | 16.48 (5.58, 27.39)** | 10.70 (-0.36, 21.75) |
| **Time** | 0.01 (-0.08, 0.09) | -0.09 (-0.23, 0.05) | -0.08 (-0.22, 0.06) | -0.07 (-0.21, 0.07) | -0.04 (-0.18, 0.10) |
| **Type of cancer treatment** |  |  |  |  |  |
| Locoregional treatment |  | Ref | Ref | Ref | Ref |
| Chemotherapy |  | -2.74 (-6.24, 0.76) | -3.10 (-6.72, 0.53) | -5.17 (-9.18, -1.16)* | -4.58 (-8.53, -0.63)* |
| Other systemic therapy^1^ |  | 4.74 (-1.54, 11.01) | 4.71 (-1.49, 10.91) | 5.45 (-1.48, 12.37) | 5.16 (-1.68, 12.00) |
| **Type of cancer treatment * time** |  |  |  |  |  |
| Local treatment * time |  | Ref | Ref | Ref | Ref |
| Chemotherapy * time |  | 0.10 (-0.08, 0.29) | 0.10 (-0.08, 0.28) | 0.09 (-0.10, 0.27) | 0.07 (-0.11, 0.25) |
| Other systemic therapy^1^ * time |  | 0.39 (0.06, 0.71)* | 0.38 (0.06, 0.71)* | 0.37 (0.01, 0.72)* | 0.35 (0.01, 0.70)* |
| **Gender** |  |  |  |  |  |
| Male |  |  | Ref | Ref | Ref |
| Female |  |  | 2.33 (-0.90, 5.55) | 0.43 (-3.37, 4.23) | 0.52 (-3.16, 4.19) |
| **Age** |  |  | 0.08 (-0.09, 0.25) | 0.02 (-0.16, 0.20) | 0.06 (-0.12, 0.23) |
| **Education** |  |  |  |  |  |
| High |  |  | Ref | Ref | Ref |
| Medium |  |  | 4.71 (1.12, 8.30)* | 4.31 (0.65, 7.97)* | 4.15 (0.61, 7.70)* |
| Low |  |  | 0.85 (-3.17, 4.88) | 0.84 (-3.21, 4.89) | 1.01 (-2.96, 4.98) |
| **Treatment completed** |  |  |  |  |  |
| Yes |  |  |  | Ref | Ref |
| No |  |  |  | 1.12 (-2.85, 5.09) | 1.02 (-2.88, 4.93) |
| **Time diagnosis to RTW ^a^** |  |  |  | -0.29 (-0.52, -0.05)* | -0.25 (-0.48, -0.02)* |
| **Extent of disease** |  |  |  |  |  |
| Local |  |  |  | Ref | Ref |
| Regional |  |  |  | 1.36 (-2.86, 5.57) | 1.35 (-2.76, 5.45) |
| Distant |  |  |  | -1.10 (-11.55, 9.34) | -1.45 (-11.40, 8.50) |
| Unknown |  |  |  | -2.47 (-6.76, 1.83) | -2.67 (-6.83, 1.50) |
| **Depressive symptoms ^b^** |  |  |  |  | 0.07 (-0.03, 0.18) |
| **Fatigue ^c^** |  |  |  |  | 0.13 (0.03, 0.24)* |
| Note: * *p* value < 0.05; ** *p* value < 0.01; *** *p* value < 0.001. Intercept, slopes and 95% confidence intervals were presented. ^1^ Other “systemic” therapy using hormonal therapy or targeted therapy, exclusively or in combination with surgery and/or radiotherapy. *RTW*, return to work; ^a^ in months; ^b^ range 0–28 ^c^range 8–56. Model 1 included *n* = 1084 (82.1%); Model 2 *n* = 1067 (80.8 %); Model 3 *n* = 1060 (80.3%); Model 4 *n* = 1024 (77.6%.); Model 5 *n* = 1023 (77.5%) of the 1320 possible person-measurement observations | | | | | |
